# MYBIOTA: A birth cohort on maternal and infant microbiota and its impact on infant health in Malaysia

**DOI:** 10.3389/fnut.2022.994607

**Published:** 2022-09-27

**Authors:** Shiang Yen Eow, Wan Ying Gan, Tiemin Jiang, Su Peng Loh, Ling Jun Lee, Yit Siew Chin, Leslie Thian Lung Than, Kang Nien How, Pui Ling Thong, Yanpin Liu, Junying Zhao, Lijun Chen

**Affiliations:** ^1^Department of Nutrition, Faculty of Medicine and Health Sciences, Universiti Putra Malaysia, Serdang, Selangor, Malaysia; ^2^National Engineering Research Center of Dairy Health for Maternal and Child, Beijing Sanyuan Foods Co. Ltd., Yinghai, Beijing, China; ^3^South Asia Branch of National Engineering Center of Dairy for Maternal and Child Health, Guilin University of Technology, Guilin, China; ^4^Research Center of Excellence, Nutrition and Non-communicable Diseases, Faculty of Medicine and Health Sciences, Universiti Putra Malaysia, Serdang, Selangor, Malaysia; ^5^Department of Medical Microbiology, Faculty of Medicine and Health Sciences, Universiti Putra Malaysia, Serdang, Selangor, Malaysia; ^6^Unit of Dermatology, Faculty of Medicine and Health Sciences, Universiti Putra Malaysia, Serdang, Selangor, Malaysia; ^7^Department of Pediatrics, Faculty of Medicine and Health Sciences, Universiti Putra Malaysia, Serdang, Selangor, Malaysia; ^8^Beijing Engineering Research Center of Dairy, Beijing Technical Innovation Center of Human Milk Research, Beijing Sanyuan Foods Co. Ltd., Yinghai, Beijing, China

**Keywords:** birth cohort study, microbiota, eczema, asthma, developmental delays, gastrointestinal disorders

## Abstract

**Background:**

The microbiota plays a key role in early immunity maturation that affects infant health and is associated with the development of non-communicable diseases and allergies in later life.

**Objective:**

The MYBIOTA is a prospective mother-infant cohort study in Malaysia aiming to determine the association between gut microbiota with infant health (temperament, gastrointestinal disorders, eczema, asthma, and developmental delays) in Selangor, Malaysia.

**Methods:**

Pregnant mothers will be enrolled in their first trimester of pregnancy, and follow-ups will be done for infants during their first year of life. Maternal-infant biological samples (blood, feces, saliva, urine, and breast milk), anthropometric, dietary, and clinical information will be collected at different time points from early pregnancy to 12 months postpartum.

**Discussion:**

This study could provide a better understanding of the colonization and development of the gut microbiome during early life and its impact on infant health.

**Clinical trial registration:**

https://clinicaltrials.gov/, identifier NCT04919265.

## Introduction

Gut microbiota plays a critical role in children's developmental pathways, especially in the first 1,000 days of life, due to the interplay between the establishment of the risks for diseases over infants' life course and infant health (1–3). Infant gut microbiota develops after birth, but the initial bacterial colonization occurs as early as when the fetus is still in the lower uterus (1). However, microbial diversity and colonization patterns vary at different time points and among infants (1, 4). More than 95% of the microbiota composition in the infant's intestinal area could be categorized into four major phyla: Firmicutes, Bacteroidetes, Actinobacteria, and Protecteobacteria (1). Two major transitions take place in the gut microbiota during infancy. The first transition characterizes the dominance of Bifidobacterium right after birth and during lactation, followed by the dominance of Bacteroidetes and Firmicutes during the weaning period, the introduction of solids foods, and continued breastfeeding practices, marking the second transition (4, 5).

Recently, increasing evidence have shown the impacts of pre-natal maternal factors on infant gut microbiota through the transmission of metabolic diseases passed from mother to child. The maternal factors are primarily associated with physical health before and during pregnancy as indicated by high pre-pregnancy body mass index (BMI) (6–8), unhealthy weight gain (6, 8), maternal exposure to medications (7–10), and gestational diabetes mellitus (GDM) (7, 8, 11). Furthermore, environmental factors involving maternal diet, including high-fat diet (7, 8, 12), smoking (10, 13), and food insecurity (14) during pregnancy are linked to the changes in infant gut microbiota. The variations in infant gut microbiota due to diet diversity among Malaysian pregnant women of different ethnic groups and the differences between Western and Eastern diets are worth investigating. Additionally, previous studies have shown that breast milk composition (4, 10, 15, 16) and maternal gut microbiota (7, 10) are linked to infant gut microbiota. Nevertheless, the composition of breast milk and gut microbiota of Malaysian women and their effects on infant gut microbiota remains unknown.

Recent discoveries have highlighted how the psychological factors of mothers, including depressive symptoms and stress, would alter gene expressions related to maternal gut homeostasis, subsequently affecting the fetal gut development and oral microbiome (17–19). However, the effects of maternal prenatal psychological factors and infant gut microbiota and immunity remain unclear. Additionally, literature on the association between maternal physical activity level and infant gut microbiota were limited to animal studies (20, 21), hence the requirement to substantiate this association *via* human studies. An extensive investigation is necessary to determine the influence of maternal pre-natal factors on infants' gut microbiome composition and diversity.

Besides pre-natal factors, accumulating studies have shown that several post-natal factors are associated with the alteration in diversity and abundance of gut microbiota in infants, including gender (22), birth order (4, 10), gestational age (4, 23), delivery mode (4, 22, 24), home settings (25), and pet rearing (24, 26, 27). Meanwhile, inconsistencies were found in associating infant growth (weight, length, and head circumference) with gut microbiota (28). Furthermore, infant feeding patterns (4, 10, 15, 22), dietary intake and diversity (29, 30) were reported as major determinants of the composition and alpha diversity of infant gut microbiota in western countries. However, despite the importance of identifying these factors, no such investigations have been conducted among Malaysian infants. Therefore, studies on infants' diets are essential due to the different dietary intake of Malaysian infants compared to those in western studies. In general, understanding the connection between pre-natal factors, post-natal factors, maternal gut microbiota, and infant gut microbiota is essential for infant health improvement and disease prevention into adulthood.

Recent studies have shown that gut microbiota is associated with temperament (31–33) and risks of gastrointestinal disorders (2, 34), eczema (35), asthma (36), and developmental delays in infants (37, 38). Temperament is a biologically-based individual differences in behavior in the form of activity, affectivity, and self-regulation, which is often used to assess a child's behaviors in early life (39). Gut microbiota influence on temperament traits warrants further inspection due to the rapid neurodevelopment at infancy (31). Thus, the current study aims to holistically understand the connection between behavior and the microbiota-gut-brain axis by studying temperament as a psychosocial variable. Furthermore, alterations in the composition and stability of infant gut microbiota lead to the development of acute and chronic gastrointestinal disorders (34). A local study showed that the prevalence of functional gastrointestinal disorders (FGIDs) among infants, including regurgitation, colic, rumination syndrome, dyschezia, functional constipation, and diarrhea, were 10.5, 1.9, 1,7, 1.3, 1.1, and 0.3%, respectively (40). Children with FGIDs were reported to have a lower quality of life, a high number of doctor visits and hospitalizations, and increased vulnerability of developing a more severe form of FGIDs and pain-related conditions in the future (41). Therefore, more research is needed to understand the association of gut microbiota and gastrointestinal disorders in infants.

Studies have shown a rapid and high diversification of gut microbiota over the first year of life in healthy children, but this is not the case for children with allergy, asthma (42, 43) and malnutrition (44). The prevalence of eczema and wheezing among Malaysian infants was 27.6 and 6.1%, respectively (45), in which the problems could be attributed to the disruption of intestinal microbiota in early life (35, 36). Besides, early life gut microbiota has been shown to be associated with the risk factor of childhood obesity, including infant's appetite traits (46). Infants who have a high abundance of the phylum Firmicutes in their gut microbiota was correlated with lower food responsiveness (46). Additionally, another study elucidated that the phylum Firmicutes was found to be abundant in the gut microbiota of obese children (47). An altered and immature gut microbiota could lead to malnutrition due to impaired energy production, vitamin biosynthesis, and immune protection (48, 49). Thus, early life intervention on immature and unstable gut microbiota is necessary to prevent the worsening of eczema, asthma symptoms, and obesity as infants grow.

Emerging evidence shows that disruption in the development of the gastrointestinal tract during the early post-natal period can affect brain development (37, 50). Impairments of developmental features relative to the child's age often refer as developmental delays (38). Poor performance on developmental domains, such as neurocognitive during childhood may affect health outcomes later in life, including poorer emotional awareness and educational attainment in later adulthood (51). The first year of life in humans is a critical period of rapid gut colonization and brain growth, but little is known about this relationship (38). Therefore, establishing a healthy gut microbiota among infants is essential to ensure optimal growth and development as well as a good quality of life.

Research on gut microbiota and the key determinants of microbial variation among Southeast Asian children remains lacking (52). Specifically, the Malaysian Working Group on Gastrointestinal Health reported a lack of research and awareness on the role of gut microbiota in early life (53). Increased awareness on the importance of gut health will help establish pre-natal and post-natal factors that promote healthy development and functioning of the immune system, gastrointestinal health, and metabolism in infants. In Malaysia, previous studies on gut microbiota mainly focused on pre-adolescent (52, 54) and pre-school children (55). While there are few local mother-infant cohort studies (45, 56, 57), none observed changes in infant gut microbiota over time.

Overall, it is essential to understand the host-microbe interactions in the first year of life since this period is crucial for infant health programming and may influence infant development with short- and long-term health consequences (1–3). The lack of evidence, especially from the Southeast Asia, calls for further studies to understand how gut microbiota in early life affects disease development (allergy, eczema, metabolic disease, and gastrointestinal disease) into adulthood. Since there is a lack of longitudinal research on infant gut microbiota in Malaysia, this cohort study aims to determine the association between gut microbiota with infant health (temperament, gastrointestinal disorders, eczema, asthma, and developmental delays) during the first year of life at different follow-ups. The specific objectives include:

1. To assess infant gut microbiota (composition, taxonomy, diversity, and abundance) during the first year of life.
2. To assess temperament, gastrointestinal disorders, eczema (presence and severity), asthma (likelihood of developing), and developmental delays of infants.
3. To determine the associations between socio-demographic, pre-natal and post-natal factors with gut microbiota in infants at 12 months.
4. To determine the associations between infant gut microbiota and infant health (temperament, gastrointestinal disorders, eczema, asthma, and developmental delays) at 12 months.

## Methods and analysis

### Study design

The MYBIOTA is a prospective cohort study involving pregnant women in the first trimester (gestational age: 8–12 weeks) and their infants up to 12 months in Selangor, Malaysia. The Standard Protocol Items: Recommendations for Interventional Trials (SPIRIT) guideline (58) was used as a reference to develop this study protocol.

### Participant selection

The sample size required for this cohort study is 225 mother-infant pairs, considering the effect size of 0.427 (59), confidence level of 95%, power of 80% and attrition rate of 29.0% (45). First, the list of private hospitals in Selangor will be obtained from the Ministry of Health. Next, eight private hospitals will be randomly selected using a computerized random number generator with an estimated participation of 30 eligible pregnant women per hospital in a month. Pregnant women aged between 18 and 45 years old in their first trimester of pregnancy who visit the selected private hospitals and plan to attend post-natal check-ups for at least 1 year in the study area will be recruited. Meanwhile, pregnant women diagnosed with immune deficiency, severe allergic conditions, psychiatric disorders, multiple pregnancies, preterm delivery (<37 weeks), a newborn with congenital abnormalities, and hospitalized in neonatal intensive care unit immediately after birth will be excluded from this study.

### Data collection timeline

**Figure 1:**
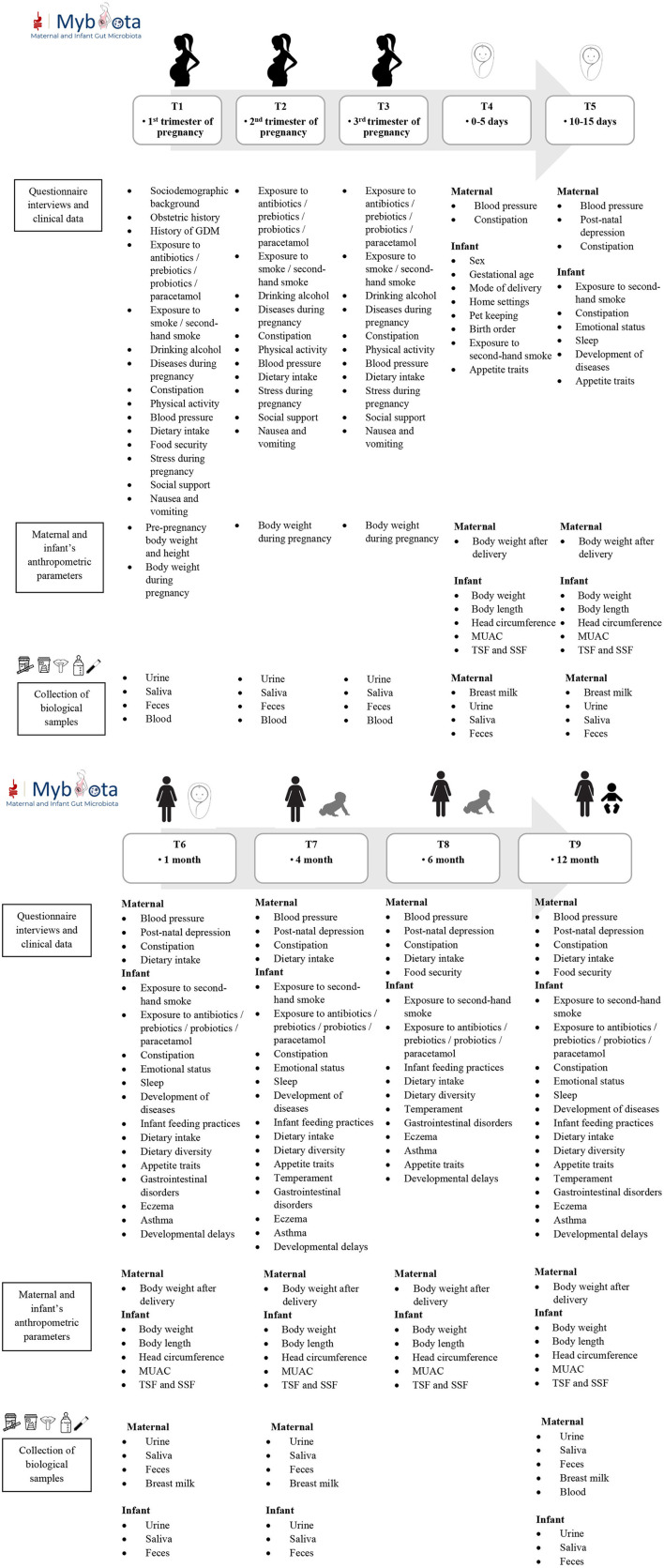
Data collection time points and sample collections of mothers and infants.

Data collection will be conducted from June 2022 to December 2024. Once the recruitment of participants begins, pregnant mothers in their first trimester will be given an information sheet explaining the purpose of the study, and those who agree to join the study are required to provide written informed consent. Data will be collected from the eligible pregnant women during their first trimester (8–12 weeks), second trimester (24 weeks), third trimester (34 weeks) and follow-up infants and mothers post-natally after birth (1–5 days after delivery), at 10–15 days, 1 month, 4 months, 6 months and 12 months of age with a total of nine follow-ups to collect mother-infant biological samples and other relevant information for the research. Types of data and samples to be collected from mothers and infants at each time point are summarized in Figure 1.

### Measurements

#### Anthropometric assessments

Maternal pre-and post-pregnancy body weight, current weight, and height will be extracted from the medical records to determine pre-and post-pregnancy BMI and gestational weight gain. Meanwhile, the infant's birth weight, current weight, length and head circumference at birth will be obtained from the child health record book. In addition, infants will be measured by researchers for their mid-upper arm circumference (MUAC), triceps skinfold (TSF) and subscapular skinfold (SSF) at 1, 4, 6, and 12 months by using a Lufkin Executive Diameter steel tape model W606PM (Cooper Hand Tools, Raleigh, North Carolina, USA) and Harpenden skinfold caliper (British Indicators Limited), respectively.

#### Biochemical assessments

A venous blood sample (6 ml) will be collected from the pregnant mothers during their routine antenatal check-ups in their first, second, and third trimester and 12 months after delivery by a certified phlebotomist. The blood will be analyzed for serum 25(OH) D level (45), iron, lipid, and metabolome profiles (60, 61) of the mothers. About 100 mg of blood sample will be mixed with 0.6 mL of 2-chlorophenylalanine-methanol solution in a 2 mL EP tube for vortex of 30 s, and then will be grinded, treated with ultrasonic, and centrifuged. Supernatant samples will be filtrated and detected with LC-MS (60). Breast milk samples are self-collected by mothers (62) at 1–5 days, 10–15 days, 1 month, 4 months, and 12 months after delivery. Human milk glycerides, phospholipids, and sphingolipids will be profiled using ultra-performance liquid chromatography/quadrupole-time-of-flight mass spectrometry [UPLC/Q-TOF-MS with electrospray ionization source (ESI)] (63). Meanwhile, the human milk oligosaccharides (HMO) will be quantified using ultra-high-pressure liquid chromatography (UHPLC) with fluorescence detection (64). Next, the membrane protein of milk fat globules will be analyzed with the high-pressure liquid chromatography Q-Exactive mass spectrometry (HPLC-QE MS) (65). Next, the immune parameters and cytokine profiles from human milk will be determined using the Luminex-approach and ELISA kits. Finally, the fatty acids composition analysis will be conducted using transmethylation techniques, capillary gas chromatography, and flame ionization (66).

#### Clinical data

Pregnant mothers will be interviewed upon recruitment using a pre-tested questionnaire. The information that will be collected includes socio-demographic data (parents' age, ethnicity, educational background, occupation, monthly household income, and marital status), obstetric history (gravida, parity, and smoking during pregnancy), and exposures (antibiotics, prebiotics, probiotics, paracetamol, and family history of diseases, exposure to smoke or second-hand smoke, alcohol consumption, constipation, emotional status, sleep, and disease development). Additionally, their prenatal blood pressure will be retrieved from the medical records.

#### Dietary assessments

Mothers will be interviewed about their dietary intake using a 24-h dietary recall at the first, second, and third trimester, and each visit at 1, 4, 6, and 12 months after birth. The dietary data will be analyzed using NutritionistPro^TM^ software (Axxya Systems, Redmond, Washington, USA) for energy and nutrients based on the Malaysian Food Composition Database (67) and the ASEAN Food Composition Database (68). Energy and nutrient adequacies are determined based on the Recommended Nutrient Intake (RNI) for Malaysians (69). Furthermore, mothers are interviewed for their infant's dietary intake using a 24-h dietary recall at 1, 4, 6, and 12 months. Infant feeding practices will also be assessed at one, four, six, and 12 months (70). The dietary diversity score will be calculated based on the infant and young child feeding (IYCF) criteria, which includes the intake of seven food groups (grains, roots and tubers, legumes and nuts, dairy products, flesh foods, eggs, vitamin A-rich fruits and vegetables, and other fruits and vegetables) (71).

#### Questionnaires

A set of validated questionnaires will be used to collect information at different time points as the following:

- Physical activity: Pregnancy Physical Activity Questionnaire (PPAQ) (72);
- Food security: Six-item U.S Department of Agriculture (USDA) Food Security Survey Module (FSSM) (73);
- Stress during pregnancy: Perceived Stress Scale (PSS)-10 (74);
- Depression: Edinburgh Postnatal Depression Scale (EPDS) (75);
- Gastrointestinal disorders of infants: Rome IV Diagnostic Questionnaire for Pediatric Functional Gastrointestinal Disorders-Toddler (R4PDQ-Toddler) (76);
- Infant temperament: Revised Infant Behavior Questionnaire (IBQ-R) (77);
- Presence of eczema in infants: five questions of the UK Working Party's Diagnostic Criteria for Atopic Dermatitis (78);
- Severity of eczema symptoms in infants: Patient-Oriented Eczema Measure (POEM) for children (79);
- Asthma symptoms in infants: Asthma Predictive Index (API) (80);
- Appetite traits: Baby Eating Behavior Questionnaire (BEBQ) (0–5 days, 10–15 days and 1 month) (81) and Children Eating Behavior Questionnaire (CEBQ) (4, 6, and 12 months) (82);
- Developmental delays: Ages and Stages Questionnaire, third edition (ASQ-3) (83).

#### Microbiota

##### Collection and transportation of samples

Saliva, urine, and feces self-collection kits will be given to mothers at each visit. Saliva sample will be collected using an oral salivary swab. Mothers are required to remove the swab from Salivette^®^ (Sarstedt, Nümbrecht, Germany) tube and gently roll her and her infant's mouth for 60 s before placing the swab into the salivette tube, per the manufacturer's instructions (65). Urine samples will be collected for metabolomic analysis using a urine collection container with boric acid as the preservative. Fresh feces from mothers and infants will be self-collected into a 5 mL feces collection tube (Sarstedt, Nümbrecht, Germany) with 2 mL of ribonucleic acid (RNA) (65). After collection of the feces sample, the cap of the feces tube will then be closed tightly followed by the shaking of tube for several times. A pad is placed in the diaper and used to collect unsoiled urine and fecal samples from the infant (65).

Mothers will be given a reminder *via* text and a tutorial video about the timing and methods of self-collection and storage of samples. They are required to notify the researchers once the self-collection and storage are done for transportation arrangements to the laboratory. All biological samples collected will be stored immediately inside a portable insulated container with ice packs at −20 °C and transported within 24-h of collection to the nutrition laboratory at Universiti Putra Malaysia for storage at −80 °C until further analysis. Once all the samples are prepared, aliquots labeled with the specific study ID number without personal information will be air freighted on dry ice and shipped to the National Engineering Center of Dairy for Maternal and Child Health, Beijing, China for further analysis.

##### Microbiome analysis

The saliva, urine, and fecal samples collected will be analyzed to examine microbiota diversity, richness, and taxonomic composition (65). First, the genomic DNA will be extracted from homogenized fecal samples using the QIAamp Fast DNA Stool Mini Kit (Qiagen, GmbH, Hilden, Germany), following the manufacturer's instruction (65). Then, the polymerase chain reaction (PCR) amplification of the V3-V4 region of 16S rRNA genes with primers Bakt_341F(CCTACGGGNGGCWGCAG) and Bakt_805R (GACTACHVGGGTATCTAATCC) (84) will be performed as the following protocol: 3 min at 95 °C, 25 cycles of 30 s at 95 °C, 30 s at 55 °C, and 30 s at 72 °C (65). Taq DNA polymerase used is the Phusion^®^ High-Fidelity DNA polymerase (M0530S, New England Biolabs, USA). Sequencing libraries will be generated using NEBNext^®^ Ultra DNA Library Prep Kit for Illumina (New England Biolabs, USA). Amplicons will be sequenced using 2 × 250 base pair (bp) paired-end protocol by Illumina HiSeq 2500. USEARCH (version 1.9) (85) will be used to quality filter, cluster, and remove chimeras from demultiplexed 16S rRNA raw sequencing data of the samples. The clustered sequences at 97% similarity level will be utilized to construct operational taxonomic units (OTU) tables and representative sequences will be assigned taxonomy from phylum to species based on the Ribosomal Database Project (RDP) 16S rRNA training set (Version 16) (86).

##### Metabolome analysis

The composition of short-chain fatty acids (SCFAs) will be identified by using the high pressure liquid chromatographic methods (Jasco Corporation, Japan) (87) and nuclear magnetic resonance (NMR) spectroscopy (88). First, the sample will be homogenized and centrifuged at low speed, and the supernatants will be filtered and mixed with phosphoric acid 0.1% as a mobile phase. Then, the mixture will be injected into the HPLC system, comprising a column Rezex™ ROA-Organic Acid H+ (8%), LC Column 300 × 7.8 mm, (Phenomenex, USA) and a UV detector at 210 nm (87). Next, the immune parameters and cytokine profiles from feces and saliva will be analyzed using the Luminex-approach and ELISA kits (87). Concentrations of the specific cytokines and the chemokines, macrophage migration inhibitory factor, macrophage inflammatory protein-1-α, monocyte chemo-attractant protein-1, immunoglobulins, and eotaxin will be determined (87). Both feces and urine samples will be further analyzed for metabolome using the NMR spectroscopy analysis (65, 88). The samples will be centrifuged, and an aqueous layer will be transferred into a 2 mL centrifuge tube. Finally, the samples will be mixed with 3-(trimethylsilyl)-1-propanesulphonic acid sodium salt (DSS) standard solution before being transferred into a tube for NMR spectroscopy analysis (65).

### Statistical analysis

The IBM SPSS Statistics software version 27 (IBM Corp., Armonk, NY, USA) will be used to analyze the data. Categorical variables will be presented in frequency and percentage, while continuous variables are presented as mean ± standard deviation (SD). The normality of continuous data will be tested using the skewness test (89). Gut microbiota will be analyzed in terms of alpha diversity, beta diversity, and differential abundance of OTUs (87). The incidence rates of asthma, eczema and gastrointestinal disorders among infants will also be calculated. Principal coordinate analysis (PCoA) plots with phylogenetic and non-phylogenetic distances will be plotted (87). One-way ANOVA with corrections (Bonferroni method) and Tukey HSD test will be carried out (89) to test for statistically significant differences in taxonomic richness, diversity measures, bacterial load, and bacterial abundances. Permutational multivariate analysis of variance (PERMANOVA) based on Bray–Curtis distances and 1,000 permutations (89) will be used to evaluate which factors are associated with the composition of the microbiota.

A generalized linear model (GLM) will be used (90) to determine the associations between predictors and gut microbiota and the association between gut microbiota and infant health after controlling for confounders. The strength and precision of each association will be determined using the relative risk (RR) with 95% confidence intervals (CI) after adjusting for potential confounders. The significance level for all statistical analyses will be set at *p* < 0.05. Meanwhile, the Bonferroni correction will be applied to counteract the effect of multiple testing and avoid multiple spurious positives (91). The alpha value will be adjusted to account for the number of comparisons performed (91).

## Discussion

Knowledge regarding the numerous contributions of the gut microbiota on human health is limited, and a lot remains to be discovered. Recent cohort studies have shown that gut microbiota is associated with temperament during infancy, which refers to biologically-based individual variation in behavior in the form of activity, affectivity, and self-regulation (31, 32). Moreover, alterations and instability of the gut microbiota composition and biodiversity during infancy could lead to the development of gastrointestinal disorders (34), eczema (35), asthma (24, 36, 42, 43), and developmental delays (37, 38).

The rising attention of good gut health development in infants to ensure optimal child development in Malaysia (52–55) necessitates this prospective cohort study. The major contributions of this study would include the prospective cohort study design and providing clarity of temporal sequence between exposures and outcome. This study involves comprehensive and prospective quantitative assessments of mothers and infants. Besides, frequent collections of urine, feces, saliva, and breast milk samples enable a holistic assessment of maternal and infant gut microbiota over time, identifying the microbiota alterations and abundance. However, one of the limitations of this study is the multiple assessments on the pregnant women during their first, second and third trimesters until 12 months postpartum, which could be potentially burdening and lead to high dropout rates. The commitment required in the collection and delivery of bio-samples in the morning may be burdensome for working mothers. These situations will be addressed by providing incentives per visit and a summary of findings and reminders about all the follow-up activities *via* phone calls and text messages. Another possible limitation is the compliance of mothers and infant sample collections (saliva, feces, urine, and breastmilk). This limitation will be overcome using user-friendly self-collection kits with clear instructions and reminders by the researchers. Furthermore, the researchers will collect samples, thus easing the burden of respondents. Besides, there are concerns on the response and social desirability bias due to the self-report nature of the questionnaire on infant health, including temperament, gastrointestinal disorders, eczema, asthma, and developmental delays. Moreover, differences in sample handling protocols, collection procedures and processing data pipelines could be the limitation of this study.

In general, the launching and implementation of this cohort is challenging, including challenges in logistic and methodological issues such as recruitment, retention, and data collection (92, 93). The common issue in the stage of launching the cohort is that it requires allocation of human resource with training provided to the researchers and enumerators to ensure proper handling of bio-samples (92, 93). During the recruitment stage, a liaison person can be assigned to build up rapport with the respondents and to perform the follow-ups in order to address respondents' concerns about their commitment required for the whole study duration, lack of interest and reluctance to enroll in the study before obtaining the husband's approval (92). Any cases involving lack of interest or husband's reluctance should be dealt with communication and inclusion of husbands in research arrangements to raise their awareness on the importance of their wife participation in the cohort. Importantly, ethical concerns involved in conducting a birth cohort must be carefully considered (93). Besides, the necessary start-up time, staff, and costs must not be underestimated (92). Moreover, additional costs relating to logistic and other expenses must be taken into consideration (92, 93).

The study findings could provide a better understanding of the colonization and development of the gut microbiome during early life. Furthermore, identifying the pre- and post-natal factors contributing to infant gut microbiota helps to fill the current knowledge gap on infant gut health. These findings could help stakeholders such as policymakers and healthcare professionals to develop appropriate educational and intervention programs to address pre- and post-natal factors that cause poor gut health among infants. Such initiatives could reduce the occurrence of preventable NCDs among infants and the financial burden on their families. Moreover, the findings of this study are useful in addressing modifiable risk factors that may impact infant gut microbiota. Suitable lifestyle, environmental, and dietary adjustments could be implemented to strengthen infants' immunity, cognition, and digestion. In summary, the gut-brain axis is a global concern and good gut health maintenance among infants is crucial in ensuring beneficial bacterial composition, diversity, and abundance in early life.

## Ethics and dissemination

The study will be carried out according to the guidelines in the Declaration of Helsinki. The ethical approval to conduct this study was obtained from the Ethics Committee for Research Involving Human Subject (JKEUPM) (Reference Number: JKEUPM-2021-418). Permission from the Director/General Manager of the selected private hospitals will also be attained prior to the data collection. The study is registered on the ClinicalTrial.gov platform, with the registration number NCT04919265 (https://www.clinicaltrials.gov/ct2/show/NCT04919265). The privacy and confidentially of the participants are protected due to the nature that the data can only be accessed by the research team and the results will be shown in group data. Written informed consent will be obtained from the participants prior to the administration of the study.

## Ethics statement

The studies involving human participants were reviewed and approved by Ethics Committee for Research Involving Human Subject, Universiti Putra Malaysia. Written informed consent to participate in this study was provided by the participants' legal guardian/next of kin.

## Author contributions

SYE, WYG, TJ, SPL, LJL, YL, JZ, and LC conceived and designed the study. SYE, WYG, TJ, LJL, YL, JZ, and LC contributed to the study design and protocol development. SYE, WYG, TJ, SPL, LJL, YSC, LTLT, KNH, PLT, YL, JZ, and LC assisted in preparation of research materials and study launch. SYE, WYG, and LJL drafted the manuscript based on the original protocol. All authors read and approved the final version of the manuscript.

## Funding

The funding of this research was supported by the Beijing Sanyuan Foods Co. Ltd.

## Conflict of interest

Authors TJ, YL, JZ, and LC were employed by Beijing Sanyuan Foods Co. Ltd. The funder had the following involvement with the study: involvement in study design, data analysis, decision to publish, or preparation of the manuscript. The remaining authors declare that the research was conducted in the absence of any commercial or financial relationships that could be construed as a potential conflict of interest.

## Publisher's note

All claims expressed in this article are solely those of the authors and do not necessarily represent those of their affiliated organizations, or those of the publisher, the editors and the reviewers. Any product that may be evaluated in this article, or claim that may be made by its manufacturer, is not guaranteed or endorsed by the publisher.
